# Gut microbiome in ADHD and its relation to neural reward anticipation

**DOI:** 10.1371/journal.pone.0183509

**Published:** 2017-09-01

**Authors:** Esther Aarts, Thomas H. A. Ederveen, Jilly Naaijen, Marcel P. Zwiers, Jos Boekhorst, Harro M. Timmerman, Sanne P. Smeekens, Mihai G. Netea, Jan K. Buitelaar, Barbara Franke, Sacha A. F. T. van Hijum, Alejandro Arias Vasquez

**Affiliations:** 1 Centre for Cognitive Neuroimaging, Donders Institute for Brain, Cognition and Behaviour, Radboud University, Nijmegen, The Netherlands; 2 Center for Molecular and Biomolecular Informatics, Radboud Institute for Molecular Life Sciences, Radboud University Medical Center, Nijmegen, The Netherlands; 3 Department of Cognitive Neuroscience, Donders Institute for Brain, Cognition and Behaviour, Radboud University Medical Center, Nijmegen, The Netherlands; 4 NIZO, Ede, The Netherlands; 5 Department of Internal Medicine and Radboud Center for Infectious Diseases, Radboud University Medical Center, Nijmegen, The Netherlands; 6 Karakter Child and Adolescent Psychiatry University Centre, Nijmegen, The Netherlands; 7 Department of Psychiatry, Donders Institute for Brain, Cognition and Behaviour, Radboud University Medical Center, Nijmegen, The Netherlands; 8 Department of Human Genetics, Donders Institute for Brain, Cognition and Behaviour, Radboud University Medical Center, Nijmegen, The Netherlands; Chiba Daigaku, JAPAN

## Abstract

**Background:**

Microorganisms in the human intestine (i.e. the gut microbiome) have an increasingly recognized impact on human health, including brain functioning. Attention-deficit/hyperactivity disorder (ADHD) is a neurodevelopmental disorder associated with abnormalities in dopamine neurotransmission and deficits in reward processing and its underlying neuro-circuitry including the ventral striatum. The microbiome might contribute to ADHD etiology via the gut-brain axis. In this pilot study, we investigated potential differences in the microbiome between ADHD cases and undiagnosed controls, as well as its relation to neural reward processing.

**Methods:**

We used 16S rRNA marker gene sequencing (16S) to identify bacterial taxa and their predicted gene functions in 19 ADHD and 77 control participants. Using functional magnetic resonance imaging (fMRI), we interrogated the effect of observed microbiome differences in neural reward responses in a subset of 28 participants, independent of diagnosis.

**Results:**

For the first time, we describe gut microbial makeup of adolescents and adults diagnosed with ADHD. We found that the relative abundance of several bacterial taxa differed between cases and controls, albeit marginally significant. A nominal increase in the *Bifidobacterium* genus was observed in ADHD cases. In a hypothesis-driven approach, we found that the observed increase was linked to significantly enhanced 16S-based predicted bacterial gene functionality encoding cyclohexadienyl dehydratase in cases relative to controls. This enzyme is involved in the synthesis of phenylalanine, a precursor of dopamine. Increased relative abundance of this functionality was significantly associated with decreased ventral striatal fMRI responses during reward anticipation, independent of ADHD diagnosis and age.

**Conclusions:**

Our results show increases in gut microbiome predicted function of dopamine precursor synthesis between ADHD cases and controls. This increase in microbiome function relates to decreased neural responses to reward anticipation. Decreased neural reward anticipation constitutes one of the hallmarks of ADHD.

## Introduction

Attention-deficit/hyperactivity disorder (ADHD) is a common neuropsychiatric disorder, characterized by symptoms of inattention and/or impulsivity and hyperactivity. ADHD has been associated with abnormalities in the monoamine neurotransmitter systems dopamine and noradrenaline [1]. Stimulant medication, for example, is highly effective in improving ADHD symptoms by inhibition of re-uptake of dopamine and noradrenaline by their transporters [2]. Moreover, brain functions linked to dopamine processing, such as reward anticipation [3], have been found abnormal in ADHD, as reflected by diminished brain responses in ventral striatum (including nucleus accumbens) in functional magnetic resonance imaging (fMRI) studies [4–7]. ADHD is highly heritable [8, 9], and genetic studies have pointed to a role of dopamine-, noradrenaline-, and serotonin-related genes in ADHD [8], but these studies showed small effects suggesting that environmental factors also play a role in the etiology of ADHD.

**Fig 1:**
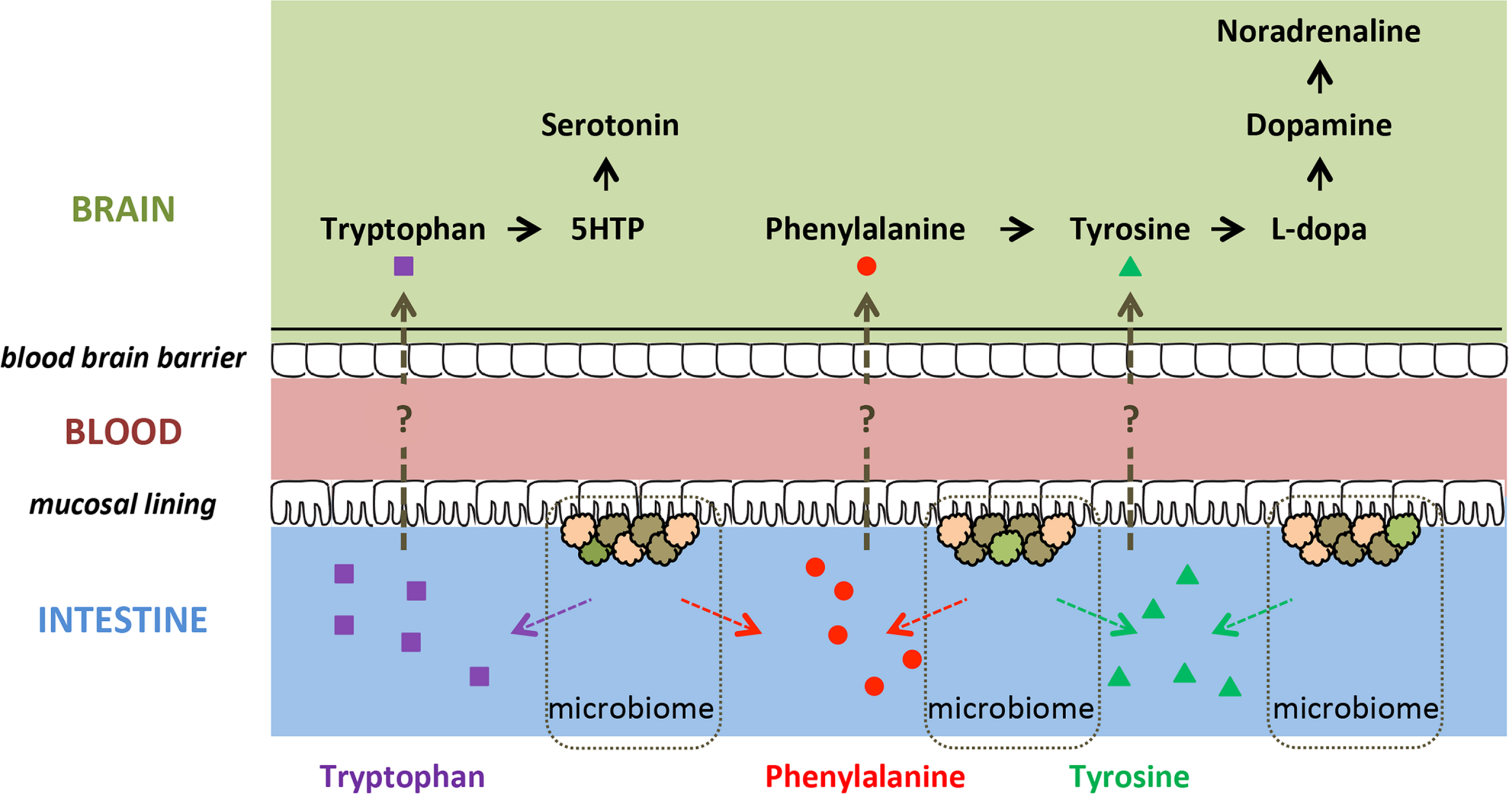
Potential routes in which precursors of monoamines could influence brain functioning. The large neutral amino acids tryptophan, phenylalanine, and tyrosine, which are absorbed in the intestine [20], are precursors of monoamines. Tryptophan and phenylalanine are essential amino acids, meaning that they cannot be synthesized by the human body itself [21]. 5-HTP = 5-Hydroxytryptophan.

Meta-analyses of non-pharmacologic treatment interventions for ADHD showed that restriction diets for ADHD patients (usually directed at eliminating potential allergens) may lead to a significant reduction in ADHD symptoms, although there is heterogeneity across studies [10–12]. Conceivably, diet might influence behavior and ADHD symptoms by affecting gut microorganisms (i.e. the gut microbiome) [13]. The gut microbiome has an increasingly recognized impact on brain functioning and behavior [14]. One proposed mechanism for the effects of gut microbiota on brain and behavior is through their ability to synthesize neurochemicals and their precursors that are analogous in structure to those of the host nervous system [15]. Precursors of monoamines involved in ADHD (i.e. dopamine, noradrenaline, serotonin; see above) are produced by several members of the gut microbiota [16–18]. These precursors (i.e. phenylalanine, tyrosine, tryptophan) might be absorbed through the intestinal epithelium, enter the portal circulation [15], and cross the blood-brain barrier; in this way, they could potentially influence host monoamine synthesis (**Fig 1**). Consequently, differences in abundance and/or metabolic activity of monoamine precursor-producing inhabitants of the gastrointestinal tract may affect monoamine-related brain functioning and behavior relevant to ADHD. Indeed, a lowered abundance of *Bifidobacterium* in infancy has been associated with increased risk of developing ADHD and Asperger syndrome in childhood in a study focusing on particular microbiota [19]. However, we were not able to identify studies that investigated the (complete) gut microbiome in relation to ADHD, and how differences in microbiome structure might affect brain functioning.

Here, we present the first microbiome study in ADHD patients versus healthy controls. We used bacterial 16S ribosomal RNA (rRNA) marker gene sequencing to characterize microbial communities in adolescents and young adults with ADHD and self-reported healthy controls. We specifically investigated the difference in relative abundance of monoamine precursor-related predicted genes between these microbiomes, and their potential effect on brain activity. We focused on the precursors in the monoamine biosynthesis, as monoamines themselves cannot cross the blood-brain barrier [20], and indeed found overabundance of 16S-based predicted bacterial gene functionality related to phenylalanine synthesis in ADHD. Next, using fMRI, we first replicated the finding of reduced reward anticipation in ventral striatum in ADHD (see above) in a partly overlapping sample of the same cohort. Finally, in the subset of participants with both microbiome and fMRI measurements, we assessed how microbiome function related to neural responses during reward anticipation. We found that reduced reward anticipation in ventral striatum was related to an increase in (predicted) bacterial functionality with regard to production of dopamine’s precursor phenylalanine, independent of diagnosis.

## Methods

### Participants

#### Microbiome sample

**Fig 2:**
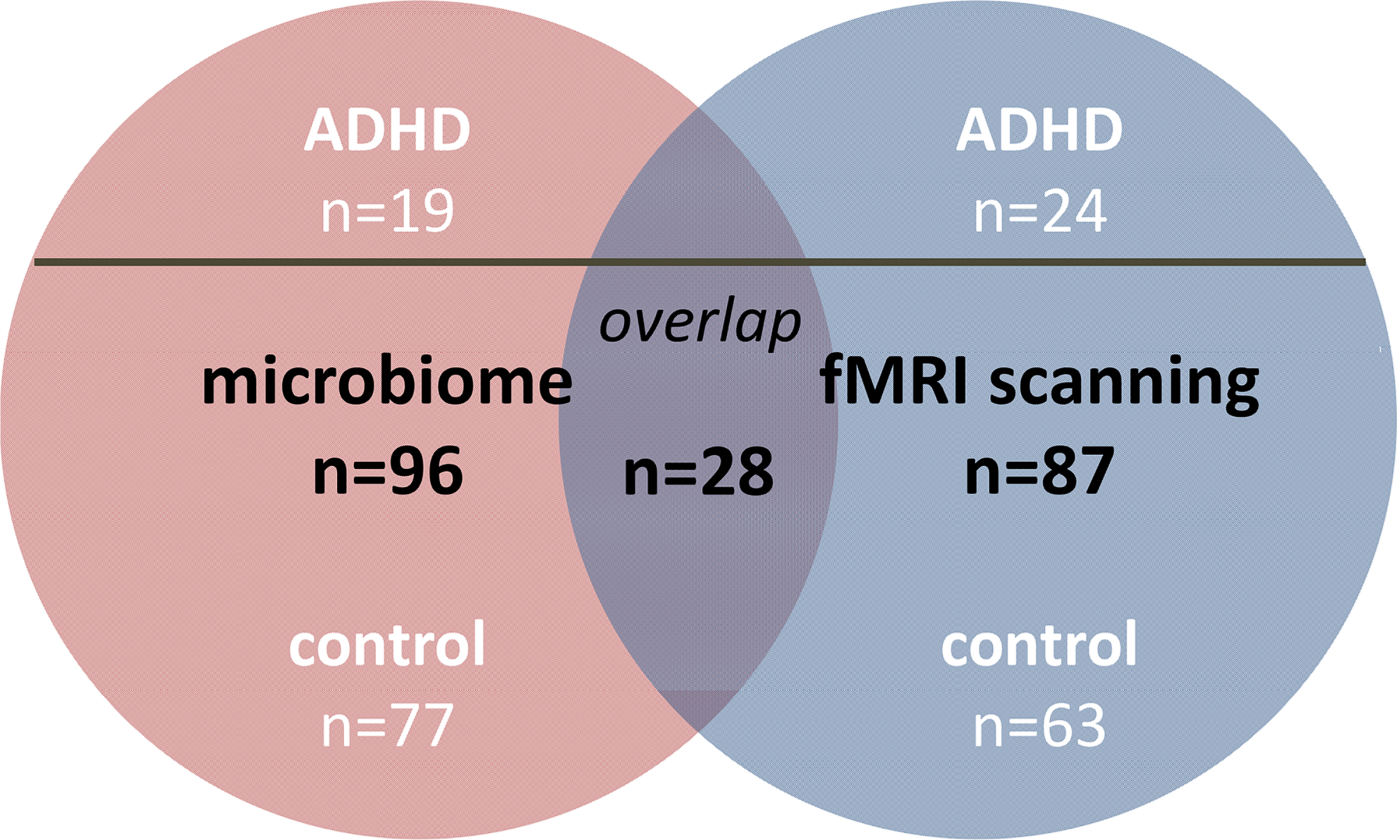
Microbiome sample, fMRI sample, and their overlap.

For the microbiome analyses, we included 96 participants, of whom 19 had been diagnosed with ADHD and 77 were healthy (**Fig 2**; **Table 1; Text A in S1 Appendix**). ADHD cases were derived from the follow-up of the NeuroIMAGE study [22] (NeuroIMAGE II) and were diagnosed based on DSM-IV symptoms using the Schedule for Affective Disorders and Schizophrenia for School-Age Children [23]. The sample of healthy individuals was compiled of two sub-samples: (i) healthy participants (n = 17) and unaffected siblings of ADHD probands (n = 21) of the ADHD cohort (NeuroIMAGE II project; not necessarily the siblings of the cases in the current sample) and (ii) self-reported healthy volunteers (n = 39) of the Brain Imaging Genetics (BIG) study [24].

**Table 1:** Descriptive characteristics of the study samples.

	Microbiome Analysis^a^			Imaging Analysis^b^			Microbiome & Imaging Analysis^c^		
	ADHD (n = 19)	Controls (n = 77)	p	ADHD (n = 24)	Controls (n = 63)	p	ADHD (n = 6)	Controls (n = 22)	p
**Age in Years (SD)**	19.5 (2.5)	27.1 (14.3)	.024	20.3 (3.7)	21.3 (3.4)	n.s.	18.6 (2.5)	21.1 (3.3)	n.s.
**BMI (SD)**	23.8 (4.1)	23.0 (3.2)	n.s.	22.8 (3.5)	22.7 (2.9)	n.s.	22.1 (4.4)	23.4 (3.7)	n.s.
**% Males**^d^	68.4	53.2	n.s.	75	61.9	n.s.	66.7	59.1	n.s.
**Mean Inattention Symptoms (SD)**	6.5 (2.1)	0.7 (1.4)^**e**^	< .001	6.2 (1.5)	0.5 (1.0)	< .001	6.0 (1.6)	0.7 (1.3)	< .001
**Mean Hyperactivity Symptoms (SD)**	4.4 (2.1)	0.6 (1.1)^**e**^	< .001	4.2 (2.4)	0.7 (1.2)	< .001	5.0 (1.4)	0.7 (1.2)	< .001
**Mean Total Symptoms (SD)**	11.0 (2.9)	1.3 (2.4)^**e**^	< .001	10.3 (3.0)	1.2 (2.1)	< .001	11.0 (1.8)	1.4 (2.4)	< .001
**Mean Number of Reads (SD)**	3893 (783)	3760 (1038)	n.s.	N/A	N/A		N/A	N/A	
**Total Number of Reads**	73969	289492		N/A	N/A		N/A	N/A	
**→Assigned at Phylum**	73876 (99.9%)	289117 (99.9%)	n.s.	N/A	N/A		N/A	N/A	
**→Assigned at Genus**	61657 (83.4%)	230370 (79.6%)	n.s.	N/A	N/A		N/A	N/A	
**Mean Number of OTU (SD)**	389 (103)	429 (157)	n.s.	N/A	N/A		N/A	N/A	
**Total Number of OTU**	7041	33048		N/A	N/A		N/A	N/A	
**Mean Shannon index** ^f^	5.3	5.2	n.s.	N/A	N/A		N/A	N/A	
**Mean Chao index** ^f^	604.5	579.7	n.s.	N/A	N/A		N/A	N/A	

^a^ Four sibling pairs were included in the ADHD group, ten sibling pairs and two trio’s in the control group. Four ADHD cases had one sibling in the control group. No BMI was available for four control subjects.

^b^ Four sibling pairs were included in the ADHD group, 13 sibling pairs and six trio’s in the control group. Nine ADHD cases had one sibling in the control group; one ADHD case had two siblings in the control group. No BMI was available for two ADHD and three control subjects. Initially, 95 participants performed the reward anticipation task during fMRI. However, four ADHD participants and four control participants were excluded from the fMRI analyses: five were excluded due to excessive (i.e. > 6 mm) movement (three ADHD + two controls), one due to an incomplete data set (ADHD), one due to too many errors (48%, control), and one due to extensive signal drop-out (control).

^c^ One sibling pair was included in the ADHD group, eight sibling pairs in the control group. One ADHD case had one sibling in the control group. No BMI was available for two control subjects.

^d^ Differences in gender between the groups were tested with chi-square or Fisher’s exact test, as appropriate. All other differences were tested with an independent t-test.

^e^ No symptoms were available for n = 39 control subjects.

^f^ Metrics for alpha diversity; reads were down-sampled to 1126 reads per sample, average of 4 trials, for the calculation of this diversity metric.

N/A: Not Available; BMI: Body Mass Index; OTU: Operational Taxonomic Unit; SD: Standard Deviation; n.s.: not significant.

#### fMRI sample

For the fMRI analyses, we included 87 participants from the above-mentioned ADHD cohort (NeuroIMAGE II project), of whom 24 had ADHD and 63 were unaffected (**Fig 2**; **Table 1; Text A in S1 Appendix**). More participants were unaffected than affected as this was a follow-up study and part of the children with ADHD no longer met the diagnostic criteria in adolescence or adulthood of the current study. Of the 63 controls, 39 participants were unaffected siblings of ADHD probands, and 24 participants were healthy controls.

Out of initial 95 participants who underwent fMRI, eight participants were excluded from analyses: one control due to significant drop-out in the back of the brain, two participants (one ADHD and one control) due to less than 10 correct trials per cell in the factorial design, five participants due to moving more than 5mm (translation; three ADHD and two controls).

For 28 included participants from the fMRI sample, group microbiome data was available: 6 patients with ADHD and 22 controls overlapped between both sample groups (**Fig 2**; **Table 1**). Of the overlapping 22 controls, 13 participants were unaffected siblings, and 9 participants were healthy controls. The regression analyses between the microbiome and the fMRI measure in this sub-sample were performed across diagnosis (n = 28); see below.

The investigation was carried out in accordance with the latest version of the Declaration of Helsinki. After complete description of the study to the participants, written informed consent was obtained (and from their parents when <18 years old). The study was approved by the regional medical ethics committee (Commissie Mensgebonden Onderzoek: CMO Regio Arnhem Nijmegen, number: NL41950.091.12).

### fMRI analysis

#### Reward anticipation task

Reward anticipation was assessed during fMRI in the context of a rewarded Stroop task (**Fig A in S1 Appendix**), adapted from a previous study [25], focusing our analyses on reward cues (high > low reward cues). Neural responses to high versus low reward cues in this task reflect motivation for monetary incentives, known to be dependent on dopamine signaling in ventral striatum [3]. Using similar tasks, reward anticipation responses were found to be reduced in patients with ADHD versus controls, and appear negatively correlated with hyperactive-impulsive symptoms in ADHD [for a review, see 6].

#### MRI data acquisition, preprocessing, and analyses

Whole-brain functional images (multi-echo) and a high-resolution anatomical scan were acquired on a 1.5T MR scanner (**Text B in S1 Appendix**). All data were pre-processed and analyzed with SPM8 (http://www.fil.ion.ucl.ac.uk/spm/). Echo combination, realignment, slice timing, co-registration, normalization, and spatial smoothing are described in **Text B in S1 Appendix**.

For each participant, the resulting pre-processed fMRI time-series were analyzed at the first level using an event-related approach in the context of a general linear model, including 24 motion parameters as regressors of non-interest (**Text B in S1 Appendix**). We performed one-sample t-tests to assess the main effects of reward anticipation (high (15 ct) > low (1 ct) reward cues) in the total sample (n = 87) as well as in the smaller sub-sample with microbiome information (n = 28). To further account for motion, we added a summary motion score for every subject in all second level analyses as covariate of non-interest (**Text B in S1 Appendix**). Statistical inference (p < 0.05) was performed at the cluster level, correcting for multiple comparisons (Family Wise Error, FWE) over the search volume, i.e. the whole brain.

We investigated the effects of diagnosis using a ventral striatum region of interest (ROI), i.e. an anatomically-defined bilateral nucleus accumbens region (**Text B in S1 Appendix**).

### Microbiome analysis

#### Microbial faecal DNA extraction

Feacal samples were collected using a standard method consisting in scooping a pea-sized piece of feces and storing it in a 50ml Falcon tube. The sample was then stored at 4°C straight after collection and at -80°C within 24 hours. Faecal genomic DNA from self-collected stool samples was isolated using the DNeasy® Blood and Tissue Kit (Qiagen, Venlo, The Netherlands) as described earlier [26]. The DNA was treated with RNase and eluted in Qiagen elution buffer AE. DNA purity and quantity were checked by spectrophotometry (ND-1000, NanoDrop Technologies, Wilmington, DE, USA).

#### 16S marker gene amplification, sequencing and data acquisition

Preparation of the amplicon pool for pyrosequencing followed established protocols [27], and is described in detail in **Text C in S1 Appendix**, where additionally sequencing and data acquisition is outlined. In short, hypervariable V3-V4 region of the 16S rRNA gene was sequenced on the 454 Life Sciences GS-FLX platform using Titanium sequencing chemistry (GATC-Biotech, Germany).

#### Microbiome sequencing data analysis

For gene sequencing analysis, a customized Python workflow based on Quantitative Insights Into Microbial Ecology (QIIME version 1.2) was adopted (http://qiime.org) [28] (**Text C in S1 Appendix**).

#### Microbiome-derived function prediction

Based on the generated 16S profiles of the gut microbiome for ADHD and control subjects for the full microbiome cohort, we predicted the presence of Kyoto Encyclopedia of Genes and Genomes (KEGG) Orthologs and subsequent functional and metabolic pathways using PICRUSt (**Text C in S1 Appendix**). Based on the KEGG (http://www.genome.jp/kegg/) pathway maps of ‘Phenylalanine, tyrosine and tryptophan biosynthesis’ (ko00400, ko00360, ko00350 and ko00380), the 17 candidate reactions/enzymes that directly result in production of phenylalanine, tyrosine, or tryptophan were selected: EC:1.3.1.43; EC:1.3.1.78; EC:1.3.1.79; EC:1.4.1.20; EC:1.4.3.2; EC:1.14.16.1; EC:2.6.1.1; EC:2.6.1.5; EC:2.6.1.57; EC:2.6.1.58; EC:2.6.1.9; EC:4.1.99.1; EC:4.1.99.2; EC:4.2.1.20; EC:4.2.1.51; EC:4.2.1.91 and EC:5.1.1.11 [29] (**Fig B in S1 Appendix**). From the relative abundances of gene functions predicted by PICRUSt we focused on candidate reactions/enzymes, by applying in-house bioinformatic (Perl-coded) scripts. Relative abundances of reactions/enzymes were calculated (**Text C in S1 Appendix**) for each individual sample based on all observed KEGG Orthologs, for each hierarchical functional KEGG level. To determine the contribution of the candidate microbial taxa, i.e. those (marginally) differing between ADHD and controls, to differences observed in the selected monoamine precursors, the PICRUSt analysis was repeated on the subset of candidate taxa only to find the taxa responsible for the observed functional effects.

#### Microbiome data analysis and statistics

Statistics on the relative abundances of selected monoamine biosynthetic pathways (K numbers: KEGG orthology groups) or taxa between sample groups was performed with SciPy (http://www.scipy.org) using a non-parametric Mann–Whitney *U* (MWU) test with Bonferroni correction for (i) multiple comparisons across all microbial taxa in all levels of phylogenetic classification (in analyses of phylogenetic composition), or (ii) across all pathways of the same KEGG hierarchical pathway level (in analyses of predicted functionality), unless stated otherwise. Relative abundance of taxa was correlated with relative abundance of enzymes/reactions (i.e. K numbers) using Spearman correlation. Subsequently, we repeated this analysis with multiple regression (in SPSS, see above) to assess the unique contribution of taxa to the predicted reaction of interest, using the 16S-based taxa as predictors and the enzymes/reactions predicted to be present in those taxa (by PICRUSt), as dependent variables. For any additional downstream sequencing-related data analysis, figures, and statistics, Microsoft® Office Excel® 2007 and GraphPad Prism version 5.03 were used.

#### Data availability

The raw, unprocessed 16S 454-sequencing reads are publicly available for download at the European Nucleotide Archive (ENA) database (http://www.ebi.ac.uk/ena) under study accession number PRJEB11512 (or secondary accession number ERP012909) [30]. The sequencing data is available in fastq-format, including corresponding metadata for each sample.

### Effects of microbiome on reward anticipation (fMRI)

In final analyses, we tested how microbiome function related to brain function. Across the whole sub-sample with both microbiome and fMRI measures available (n = 28), we assessed the effects of relative abundance of a candidate predicted microbiome-derived enzyme/reaction as covariate of interest on whole-brain reward anticipation responses (in SPM) with the reward anticipation (high > low) images (pFWE < 0.05, cluster-level, small volume: bilateral nucleus accumbens region from the Hammersmith atlas (**Text B in S1 Appendix**).

We also performed multiple linear regression with the ventral striatum ROI betas for reward anticipation (see above) as dependent variable and the microbiome measure as well as ADHD diagnosis, age, gender, and stimulant medication use as predictors (**Text B in S1 Appendix**).

## Results

### Demographics

The ADHD cases and healthy participants did not differ in BMI and gender in the three groups (microbiome, fMRI, and their overlap) (**Table 1**). No differences in age were present for patients and healthy persons in the fMRI samples, but for the microbiome analyses, controls were older (**Table 1**). This was due to the fact that the 39 BIG participants were older (33.1 years ± 17.7 SD). Controls from the NeuroIMAGE II study (21 unaffected siblings [22.3 years ± 3.7 SD], and 17 healthy controls [19.1 years ± 3.2 SD]) did not differ from the cases (t(55) = -1.46, p = .15). Consequently, the functional (PICRUSt) analyses below were performed with and without the older BIG controls to balance power and homogeneity of the sample.

### Gut microbiome taxa

**Fig 3:**
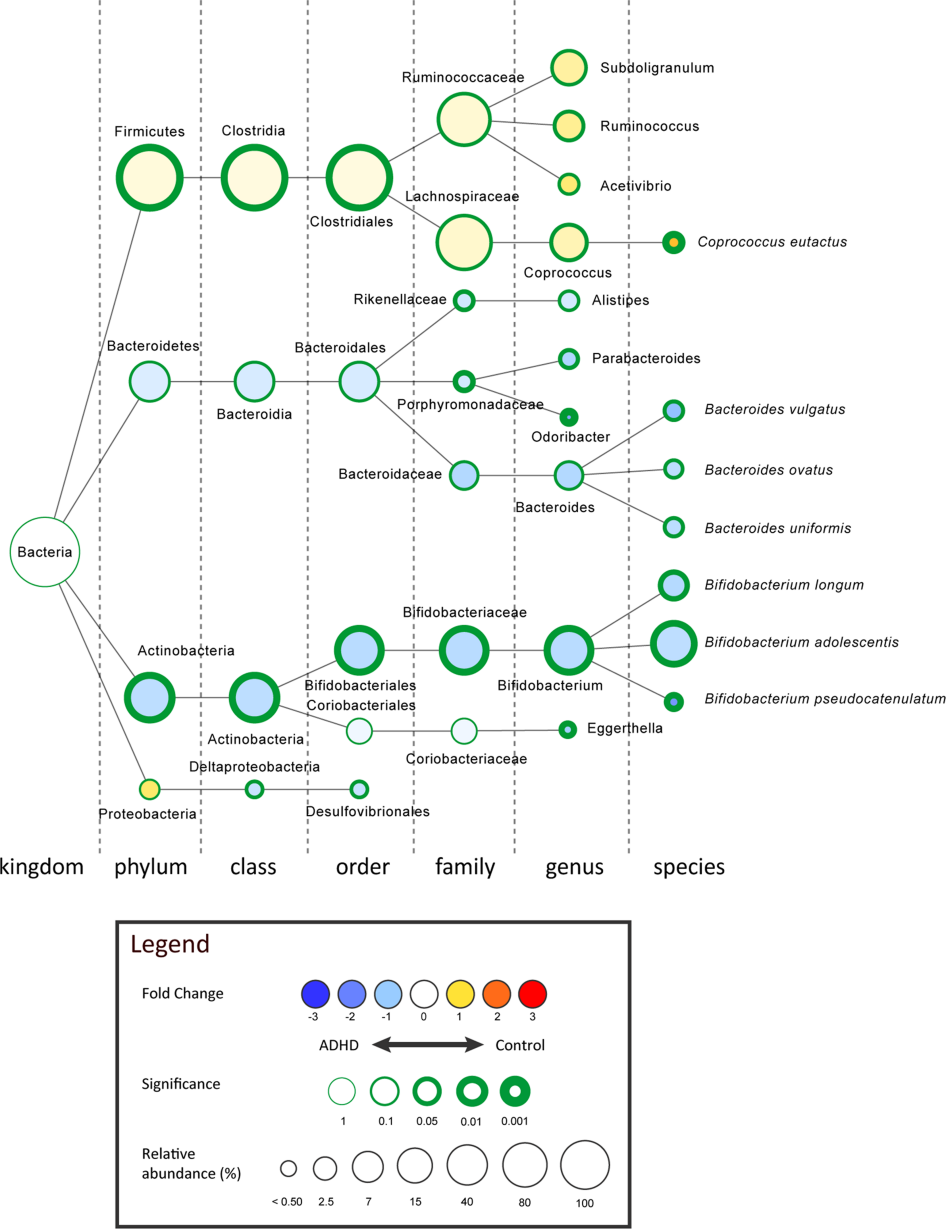
The strongest differentially abundant microbial taxa for ADHD cases (n = 19) versus healthy controls (n = 77), shown in the graphical Cytoscape visualization [32]. Nodes represent taxa (node size represents average relative abundance, for both experimental groups combined), edges (dashed lines) link the different taxonomic levels. The weighed fold-change (node color) is calculated as the 2log of the ratio of the relative abundance between control and ADHD (0 = no difference between genotypes, 1 = twice as abundant in control, etcetera). In other words: yellow to red indicates an overrepresentation in control, hence an underrepresentation in ADHD, and vice versa for light- to dark blue. The significance (node border width) is expressed as the p-value of a Mann–Whitney *U* test, uncorrected for multiple comparisons.

For sample characteristics, diversity metrics and read counts, see **S1 Table** and **Text D in S1 Appendix**. The overall make-up of the 96 microbiomes consisted of bacteria predominantly from the phyla Firmicutes (77.92%), Actinobacteria (15.68%) and Bacteroidetes (6.05%) (**Fig 3; Fig C in S1 Appendix; S2 Table**). We found an increase of Actinobacteria (controls: 14.08% to ADHD: 22.14%; p = 0.002, uncorrected), which seemed to occur mainly at the expense of Firmicutes (controls: 79.80% and ADHD: 70.29%; p = 0.001, uncorrected), as Bacteroidetes (and other phyla) did not differ significantly (controls: 5.74% to ADHD: 7.29%; p = 0.166, uncorrected) in relative abundance between healthy participants and those with ADHD (**S2 Table;** MWU, multiple comparisons corrected p-value threshold = 0.00017). Interestingly, in more taxonomic detail, within the phylum Actinobacteria, the genus *Bifidobacterium* was significantly increased in ADHD cases (controls: 12.66% to ADHD: 20.47%; p = 0.002, MWU, uncorrected) (**Fig 3**). *Bifidobacterium* dominates the gut early in life, and slowly decreases in relative abundance during ageing [31]. In order to exclude an age-driven shift in *Bifidobacterium*, we defined an age-matched subsample for our microbiome cohort (**Text D in S1 Appendix**). Using 15 pairs of ADHD cases and age-matched controls we find a similar rise in *Bifidobacterium* in ADHD-affected individuals (controls: 13.77% to ADHD: 18.90%) (p = 0.034, MWU, uncorrected). Furthermore, the order Clostridiales, within the phylum Firmicutes, was found to be decreased in ADHD cases (controls: 77.37% to ADHD: 69.02%; p = 0.003, MWU, uncorrected) and best explains the observed drop in Firmicutes, but no specific taxonomical entity belonging to the order Clostridiales was found to be responsible for this effect (**Fig 3**). In conclusion, the genus *Bifidobacterium* shows the most (statistically) strong and taxonomic most specific change as effect of ADHD status.

We selected five candidate taxa with the greatest difference between ADHD and controls to be used in our subsequent analyses of metabolic potential (by PICRUSt): Clostridiales (order), Rikenellaceae (family), Porphyromonadaceae (family), *Bifidobacterium* (genus) and *Eggerthella* (genus).

### Hypothesis-driven analysis of gut microbiome metabolic potential

We predicted bacterial gene function using PICRUSt, focusing on pathways involved in the synthesis of phenylalanine, tyrosine, and tryptophan, which can serve as precursors of human dopamine, noradrenaline and serotonin (**Fig 1**).

**Fig 4:**
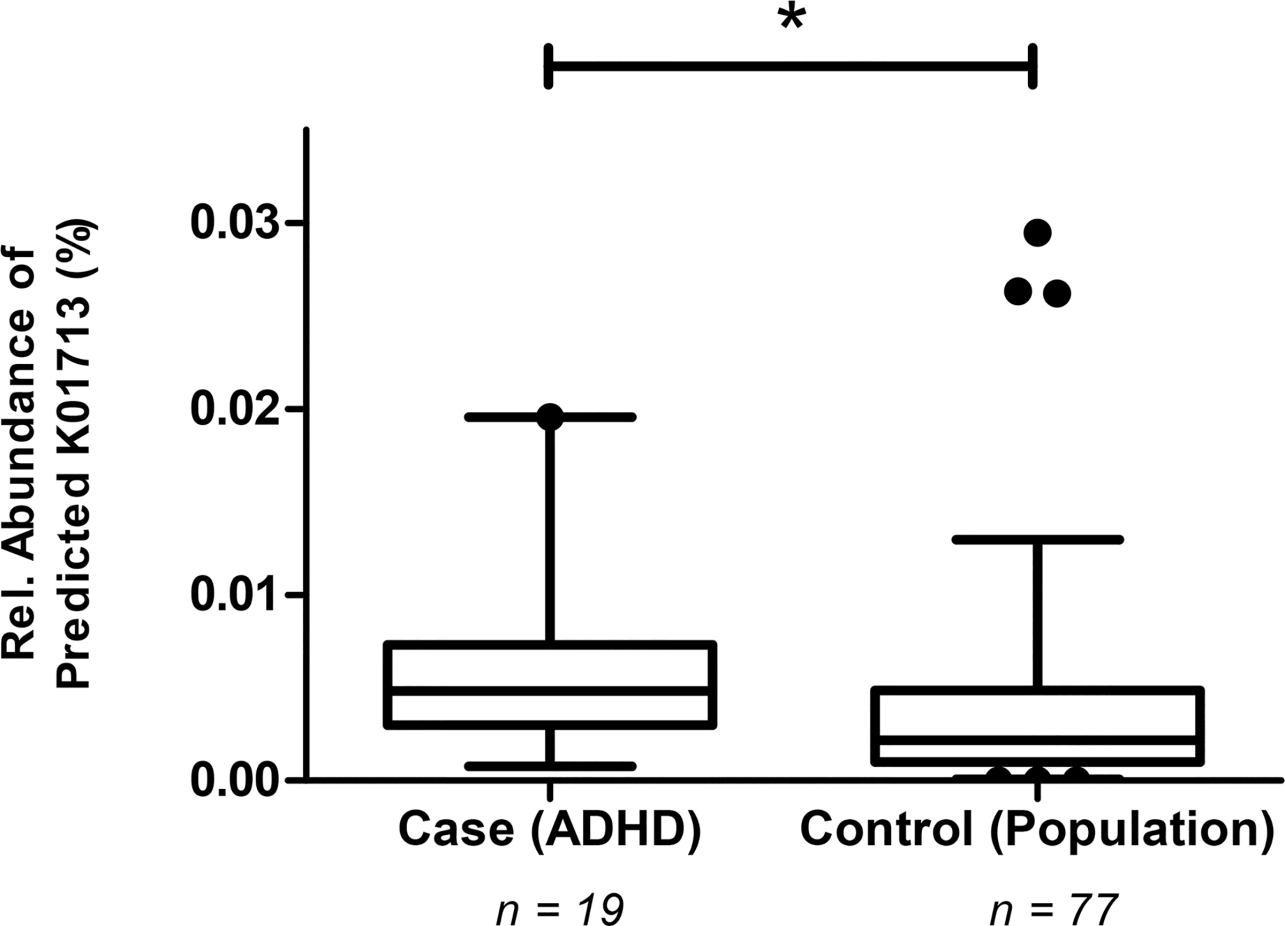
The ADHD microbiome contains significantly increased levels of predicted cyclohexadienyl dehydratase (CDT; KEGG Ortholog K01713; EC:4.2.1.51), responsible for phenylalanine synthesis (Fig B in S1 Appendix). This analysis is based on functional predictions deriving from 16S profiles of the microbiome, as performed by PICRUSt [33]. Box plots represent the relative abundance of predicted CDT, with 5–95% percentile whiskers (dots represent outliers). The significance was tested with a non-parametric MWU (* p = 0.038), Bonferroni-corrected for 15 K numbers identified.

Relative abundances of the 17 candidate reactions/enzymes (pathways) that are directly involved in the production of phenylalanine, tyrosine, or tryptophan (**Fig B in S1 Appendix**) were predicted based on the total gut microbiome of healthy controls and ADHD cases. For these 17 *a priori* selected candidates, 15 K numbers could be identified in the microbiome (**S3 Table**). One predicted enzyme, cyclohexadienyl dehydratase (CDT; KEGG Ortholog K01713; EC:4.2.1.51), was found to be significantly more abundant (on average, 150% more than in controls) in the microbiome of ADHD cases (p = 0.038 by MWU, Bonferroni-corrected for 15 K numbers identified) (**Fig 4; S3 Table**). Moreover, CDT ranked among the top ~1% of a total of 7545 reactions (when we sort our reaction *p-*values from low to high (**S3 Table**). Similar results of ADHD cases versus controls were obtained with the sample groups matched for age (i.e. without the older BIG controls), though significance is lost (p = 0.085, Bonferroni-corrected). Using logistic regression to control for age (and gender; **Text D in S1 Appendix**) the results in the age-matched sample (i.e. without the BIG controls) hint to the fact that the change in ADHD for K01713 is a significant effect (p = 0.024), but the marginal effect in the complete sample (p = 0.070) shows that our results cannot be completely disconnected from the age confounder present in the cohorts studied (**Text D in S1 Appendix**).

To assess which of the five taxa differing between cases and controls (**Fig 3**) contributed most to the observed difference in the predicted enzyme CDT, we repeated the functional (PICRUSt) analysis for these candidate taxa only: Clostridiales (order), Rikenellaceae (family), Porphyromonadaceae (family), *Bifidobacterium* (genus) and *Eggerthella* (genus) (**Fig 3**). Differences in relative abundance of the genus *Bifidobacterium* uniquely contributed to the observed differences in the predicted phenylalanine pathway enzyme CDT in a multiple regression analysis (p < 0.001). The same conclusion was drawn from the fact that in the cohort *Bifidobacterium* contributed 99.9% of the predicted CDT (K01713) counts relative to the predicted CDT counts based on PICRUSt analysis of the entire microbiome (n = 16,340; **Text D in S1 Appendix**).

### fMRI: Effects of reward anticipation and diagnosis

**Fig 5:**
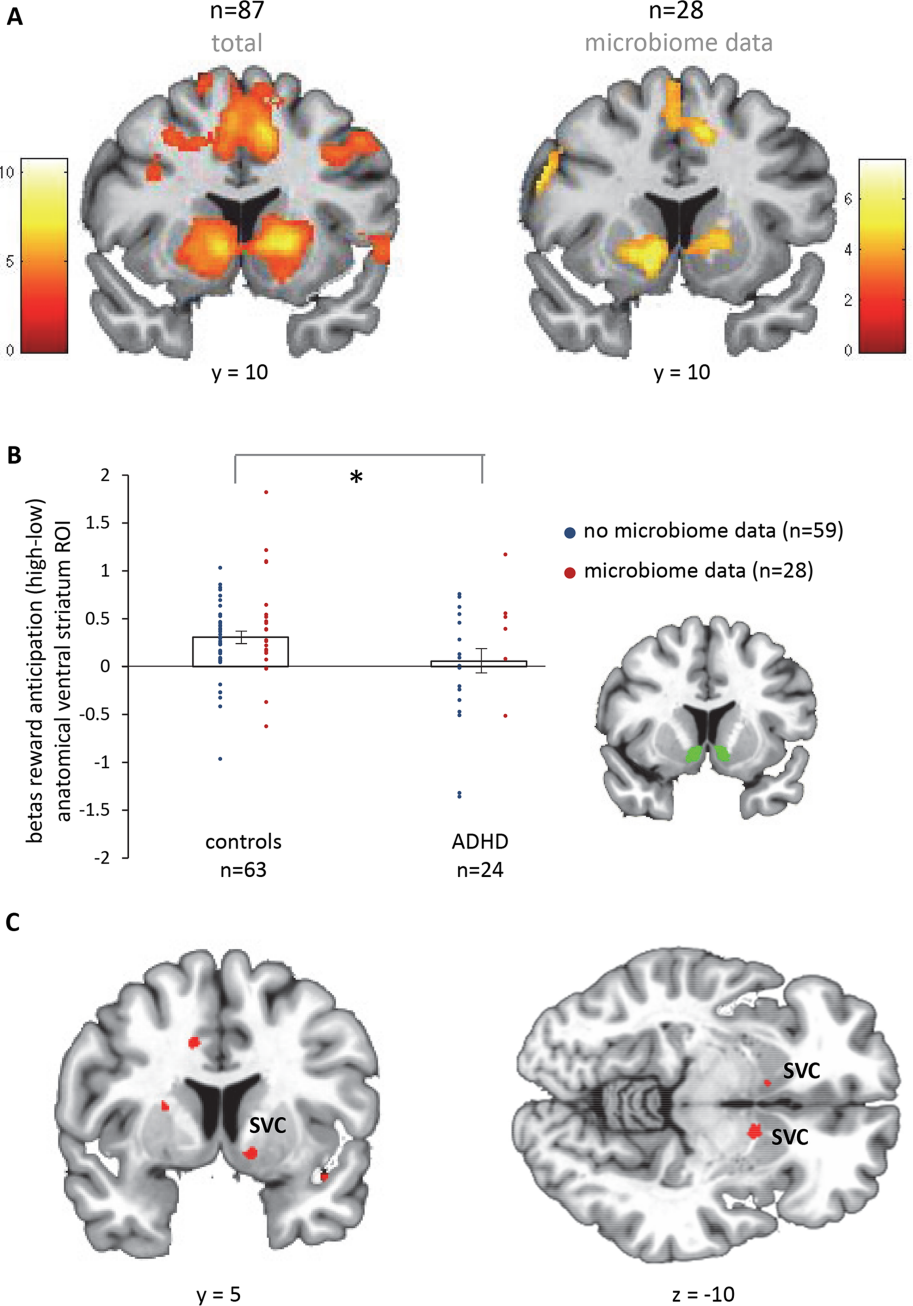
fMRI results. **A**. Main effect of reward anticipation, cluster-level corrected at the whole-brain level (pFWE < 0.05). Color bars reflect T-values. **B**. Diagnosis effects in the anatomical region of interest (ROI) of the ventral striatum. **C**. Negative correlation of the microbiome function CDT (see **Fig 4**) with reward anticipation responses across the whole-brain (n = 28), intensity threshold at p < 0.001 uncorrected (T = 3.45). The clusters in bilateral ventral striatum (x = -11, y = 11, z = -9, cluster size = 8, p(FWE, cluster) = 0.024; x = 11, y = 6, z = -11, cluster size = 2, p(FWE, cluster) = 0.036) are significant after correcting for multiple comparisons across the search volume (cluster-level pFWE < 0.05, SVC), i.e. the anatomically defined ventral striatum shown in panel B. SVC = small volume correction. * indicates p < 0.05.

In a partly overlapping sample of the same cohort, we tried to replicate reduced reward anticipation in ADHD versus controls (see Introduction). Across the whole sample, reward anticipation (high (15 ct) > low (1 ct) cues) elicited brain responses in the striatum and the occipital, premotor, and frontal cortices (pFWE < 0.05, whole-brain, cluster-level correction) in our total fMRI sample (n = 87) as well as the sub-sample with microbiome data (n = 28) (**Fig 5A; Text E in S1 Appendix**). Taking the anatomically-defined ventral striatal ROI, we indeed found decreased ventral striatal responses for reward anticipation in patients with ADHD versus controls (t(85) = 2.1, p = 0.038) (**Fig 5B**). This difference was not significant in the sub-sample with microbiome data (t(26) = 0.2).

### fMRI: Effects of the microbiome on reward anticipation

Finally, we assessed how the functional microbiome measure found to be different for ADHD relative to controls (i.e. predicted CDT), would relate to neural reward anticipation in the sub-sample with both microbiome and fMRI data. Across the whole sub-sample (n = 28), independent of diagnosis, we observed a negative association (whole-brain) of the relative abundance of predicted CDT with reward anticipation responses in bilateral ventral striatum (**Fig 5C**). This effect was also significant in the ventral striatal ROI analysis (standardized beta: -0.48, p = 0.032), when including possible confounding factors (ADHD diagnosis, age, and gender) in the analysis. Also, when adding stimulant use (duration times dose) to control for long-term medication effects, predicted CDT relative abundance was still significantly associated with reward anticipation responses in ventral striatum (standardized beta: -0.42, p = 0.048).

## Discussion

This is the first study investigating microbiome differences between ADHD cases and controls. Observed differences in taxa in this exploratory study were strongest for the phylum Actinobacteria, which was more abundant in cases, apparently at the expense of Firmicutes, for which abundance was lower in cases; uncorrected for multiple comparisons. In addition, we describe for the first time effects of a genetically encoded capacity for production of monoamine precursors in the gut microbiome of ADHD: the enzyme CDT, involved in the synthesis of a dopamine precursor (phenylalanine), was predicted to be significantly more abundant in the microbiome of ADHD cases compared to healthy individuals (corrected for multiple comparisons). This effect appeared dependent on age, although significant effects were obtained with age-matched samples. The genus *Bifidobacterium* (within the phylum Actinobacteria)–also more abundant in (age-matched) ADHD versus controls (uncorrected for multiple comparisons)–appeared to be solely responsible for this predicted enzyme CDT increase. Across the sample (i.e. independent of diagnosis), the abundance of predicted CDT genes from the microbiome correlated negatively with bilateral ventral striatal BOLD responses for reward anticipation (corrected for multiple comparisons in an anatomically-defined search volume), typically reduced in ADHD [4–7] and replicated here.

Pärtty and colleagues [19] recently used markers for specific bacterial species to show that decreased numbers of *Bifidobacterium* (e.g. *Bifidobacterium longum*) in 3 and 6 months old children (treated with either a *Lactobacillus*-based probiotic or a placebo) predicted ADHD or Asperger syndrome manifestation at age 13 years [19]. At the time of diagnosis (13 years old), they did not observe any significant difference in the assessed taxa, including *Bifidobacterium*, between ADHD and controls. This difference with our marginally significant result of increases in *Bifidobacterium* in ADHD might be explained by (i) our more sensitive method (16S marker gene sequencing versus PCR), and (ii) larger sample size (19 ADHD and 77 controls versus 6 ADHD/Asperger and 69 controls) or (iii) differences introduced by experimental procedures or between cohorts. The observed differences in *Bifidobacterium* genus between controls and adolescents/adults with ADHD (current study) or infants developing ADHD later in life [19]–in opposite direction–require formal replication in longitudinal studies with larger samples. Decreased abundance of *Bifidobacterium* in early infancy versus increased abundance in early adulthood might reflect delayed gut microbiome maturation in ADHD, as *Bifidobacterium* is known to decline with older age [31]. Pärtty and colleagues [19] found that *Bifidobacterium* in infancy predicted ADHD and Asperger syndrome. Changes in microbiome composition have indeed consistently been found in autism spectrum disorder [34]. Although participants with DSM-IV defined Autistic Disorder and other neuropsychiatric disorders were excluded, we cannot be certain that the current findings are specific for ADHD.

*Bifidobacterium* was responsible for increases in the predicted function of CDT (K01713)—involved in the synthesis of phenylalanine (**Fig B in S1 Appendix**)—in ADHD cases versus controls. Phenylalanine is an essential amino acid, which cannot be synthesized by humans and has to be absorbed from the gut[21]. Phenylalanine can cross the blood-brain barrier and is the precursor of dopamine and noradrenaline [20] (**Fig 1**). These neurotransmitters are highly affected in ADHD, nevertheless, the exact mechanisms of involvement are still ambiguous [35–37]. Predicted CDT correlated negatively with ventral striatal responses during reward anticipation. Striatal reward anticipation responses are modulated by dopamine and remediated by methylphenidate [3, 38]. Our findings suggest that gut microbial-induced levels of CDT correlate functionally with available levels of the dopamine precursor phenylalanine, which could potentially be a risk factor for disturbed dopamine signaling and reduced brain reward responses. Indeed, high levels of phenylalanine have been linked to ADHD symptoms [39]. However, this association was found in phenylketonuria, a disorder in which phenylalanine cannot be converted to tyrosine, which results in toxic build-up of phenylalanine in the brain. Two other studies, using an overlapping sample, have found decreased blood plasma concentrations of phenylalanine in ADHD [40, 41] and a more recent and larger study did not observe an association between peripheral (i.e. blood and urine) levels of phenylalanine and ADHD [42]. Future studies should link the presently observed increases in predicted microbiome-derived phenylalanine to peripheral levels of phenylalanine and study how this relates to brain function.

Moreover, the mechanism by which the increased predicted CDT function (and speculatively, increased phenylalanine) would result in decreased striatal BOLD responses for reward anticipation remains to be investigated. Many different routes for a potential causal relationship are possible. Microbial phenylalanine could be absorbed in the blood stream, cross the blood-brain barrier, and influence dopamine synthesis (**Fig 1**) positively or negatively (by inhibiting tyrosine hydroxylase) [43]. Alternatively, on the host side, altered blood plasma levels of phenylalanine (or its derivate tyrosine) could have an effect on the synthesis of neuromodulators other than dopamine, e.g. by competing at the blood-brain barrier with tryptophan (precursor of serotonin) or by conversion to trace amines [20, 44, 45]. In addition, yet unknown interactions of multiple bacterial groups involved in the production of neuroactive substances might affect host neurophysiology within the gastrointestinal tract [15]. Nevertheless, our observed relationship between the microbial phenylalanine pathway and neural responses for reward anticipation, known to depend on phenylalanine’s derivate dopamine, may argue for a dopaminergic effect at the level of the brain instead of the gut. Future research should confirm this and show specificity for microbial functions, as well as brain regions and associated functions.

Our study should be viewed in the context of its strengths and limitations. Obvious strengths include the 16S microbiome analysis (instead of preselected candidate taxa), larger sample size than previous studies in ADHD [19], and our mechanistic, hypothesis-driven approach in terms of predicted enzymes (function analysis) as well as their link to brain functioning using fMRI. As for limitations, a microbiome shotgun sequencing method preferably combined with a proteomics or metabolomics approach instead of the 16S marker gene method might have allowed us to make more solid claims about microbial biological function. Second, about 25% of our control participants were siblings of ADHD cases (see Table 1), and another sub-sample of the control group did not undergo clinical screening for ADHD (the BIG sample). However, in both cases this would be more likely to cause an underestimation of differences between ADHD cases and controls. Third, the control group was significantly older than the ADHD cases. Consequently, the age confounder in our study hampered us from attributing the significant change in predicted CDT exclusively to ADHD. Nevertheless, our study suggests that the change in ADHD for the CDT enzyme (K01713) is a significant effect, with subject age having an important contribution. This might in part be explained by the explicit nature of ADHD for which it is recognized that its prevalence diminishes with age [46]. Importantly, the gut-microbiome-brain association between predicted CDT and reward anticipation fMRI responses was observed independent of age. Fourth, it is well known that diet affects microbiome structure/stability [13]. In this respect, having a substantial part of the control group consisting of unaffected siblings of ADHD cases (21/77), presumably living in the same household with similar diets, should be viewed as a strength of this study. Importantly, we did not observe any differences in body mass index between the groups. Fifth, our gut-microbiome-brain association was found independent of, or actually controlled for, ADHD diagnosis. Hence, we can only conclude that functional differences found between ADHD and controls at the microbiome level are related to neural effects *across subjects*. ADHD status-specific conclusions about this gut-brain relation might be made in future studies with larger group sizes. Finally, given the generally observed beneficial effects of *Bifidobacterium* [47, 48], it remains to be resolved in subsequent studies whether our observed effects are a consequence or, perhaps, a compensatory, effect of ADHD status. Considering these uncertainties, our novel functional gut-brain approach provides many leads for new research, but caution should be taken to translate these findings to non-pharmacological intervention strategies in ADHD.

## Conclusions

This is the first study demonstrating differences in the gut microbiome between patients with ADHD and healthy individuals, using a comprehensive 16S microbiome analysis, and showing–if anything–an increase in the genus *Bifidobacterium*. This increase was associated with significantly enhanced predicted biosynthesis potential of a dopamine precursor in the gut microbiome of ADHD patients versus controls, which was linked to altered reward anticipation responses in the brain, a neural hallmark of ADHD. With this mechanistic approach, we hypothesize that presumed differences in dopamine precursor production at the gut microbiome level in ADHD might be related to dopamine disturbances at the neural level associated with reduced brain reward responses. This study highlights the importance of investigating the functional effects of microbiome differences in neuropsychiatric disorders.

## Supporting information

S1 Table Microbiome descriptives. (XLSX)

S2 Table Relative abundance taxa. (XLSX)

S3 Table Relative abundance pathways. (XLSX)

S1 Appendix Additional figures and text. File including Figures A-C (Reward anticipation task; Monoamine precursor biosynthesis pathways; Intestinal microbiome composition) and Text A-F (Cohorts; fMRI parameters and analyses; Microbiome sequencing and analyses; Additional information microbiome; fMRI main effects of reward anticipation; References).
(PDF)
