# Supplementary material for: Gut microbiome in ADHD and its relation to neural reward anticipation
Source: PLoS One. 2017 Sep 1;12(9):e0183509. doi: 10.1371/journal.pone.0183509 (PMC5581161; doi:10.1371/journal.pone.0183509)
Supplement: S1 Appendix — File including Figures A-C (Reward anticipation task; Monoamine precursor biosynthesis pathways; Intestinal microbiome composition) and Text A-F (Cohorts; fMRI parameters and analyses; Microbiome sequencing and analyses; Additional information microbiome; fMRI main effects of reward anticipation; References). (PDF) [file pone.0183509.s004.pdf]

S1 Appendix
(see also separate S1-3 Tables)

Figures

a. Fig A: Reward anticipation task
b. Fig B: Monoamine precursor biosynthesis pathways
c. Fig C: Intestinal microbiome composition

Text A: Cohorts

1. NeuroIMAGE II cohort
2. BIG cohort
3. Inclusion criteria microbiome part

Text B: fMRI parameters and analyses

1. fMRI data acquisition
2. fMRI preprocessing
3. fMRI statistical analysis
4. Region of Interest (ROI)
5. Psychostimulant predictor in multiple regression

Text C: Microbiome sequencing and analyses

1. 16S marker gene amplification, sequencing and data acquisition
2. Microbiome sequencing data analysis and BioIT workflow
3. Microbiome-derived function prediction

Text D: Additional information microbiome

1. Gut microbiome sample characteristics
2. Age-matched sub-sample of ADHD cases and controls
3. Gut microbiome metabolic potential: logistic regression including age
4. Relation between taxa and reactions

Text E: fMRI main effects of reward anticipation

Text F: References in S1 Appendix

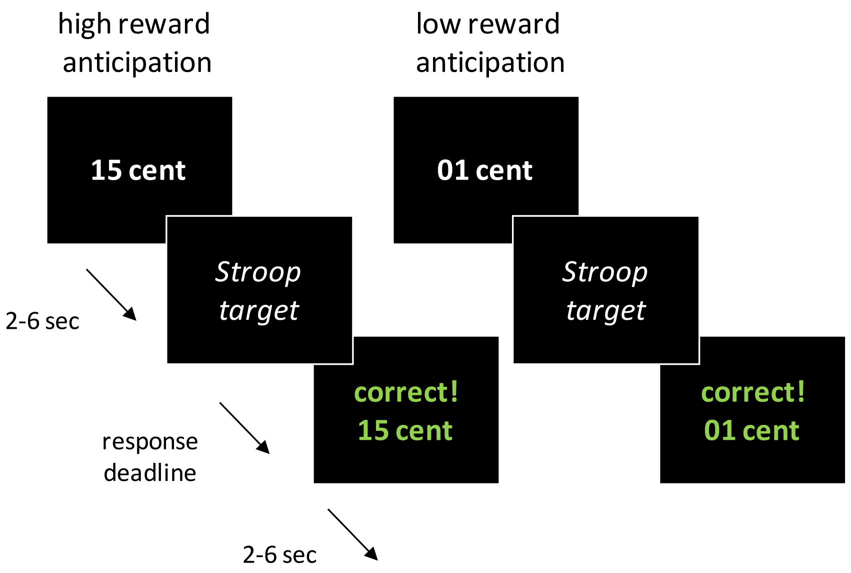

Figure A. Reward anticipation task.
Reward anticipation was assessed during fMRI in the context of a rewarded Stroop task. All trials began with a cue predicting high (15 cents) or low (1 cent) reward for correct performance on the target. The targets were arrow-word Stroop-like stimuli, with the word “LEFT” or “RIGHT” (relevant dimension) positioned in a left- or right-pointing arrow (irrelevant dimension). Participants responded to the target word using two button boxes, pressing the left button box with the left thumb or the right button box with the right thumb. The direction denoted by the word was either congruent or incongruent with the direction indicated by the arrow. Here, we focused our analyses on the reward cues (i.e. reward anticipation: high > low reward cues) preceding the targets. The task resembled a Stroop paradigm used previously [1], except that the ‘information cues’ were excluded from the current paradigm, the inter-stimulus intervals were greater (2–6 sec), and the participants received direct feedback on whether the response was correct (+ 1 or + 15 cent earned), incorrect (0 cent earned), or too late (0 cent earned). Reward cues and target congruency were equally distributed across the 120 trials (duration of 20 min).
Two practice blocks preceded the actual experiment: one to familiarize the participants with the Stroop task (40 trials) and one to familiarize the participants with the actual task including the reward cues (40 trials). The practice blocks were used to set the initial response window in the next part of the experiment, which was the average response time (RT) for trials responded to correctly per trial type. In the main experiment, reward was obtained only when an answer was correct and occurred within this response window determined individually for each participant. The initial response windows were adapted throughout the main experiment: after a correct response that was on time, 10 ms was subtracted from the response window for that trial type, and after a response that was too late, 25 ms was added to the response window for that trial type. Hence, frequency of reward receipt did not vary with difficulty and was similar across participants. After every 30 trials, participants were informed about the total amount of reward obtained at that point in a 15 seconds break. The reward money (mean ADHD: 5.94 EUR, SD: 1.15 EUR; mean controls: 5.91 EUR, SD: 0.80 EUR; $t(85)=-0.1$, ns) was added to the participants’ compensation.

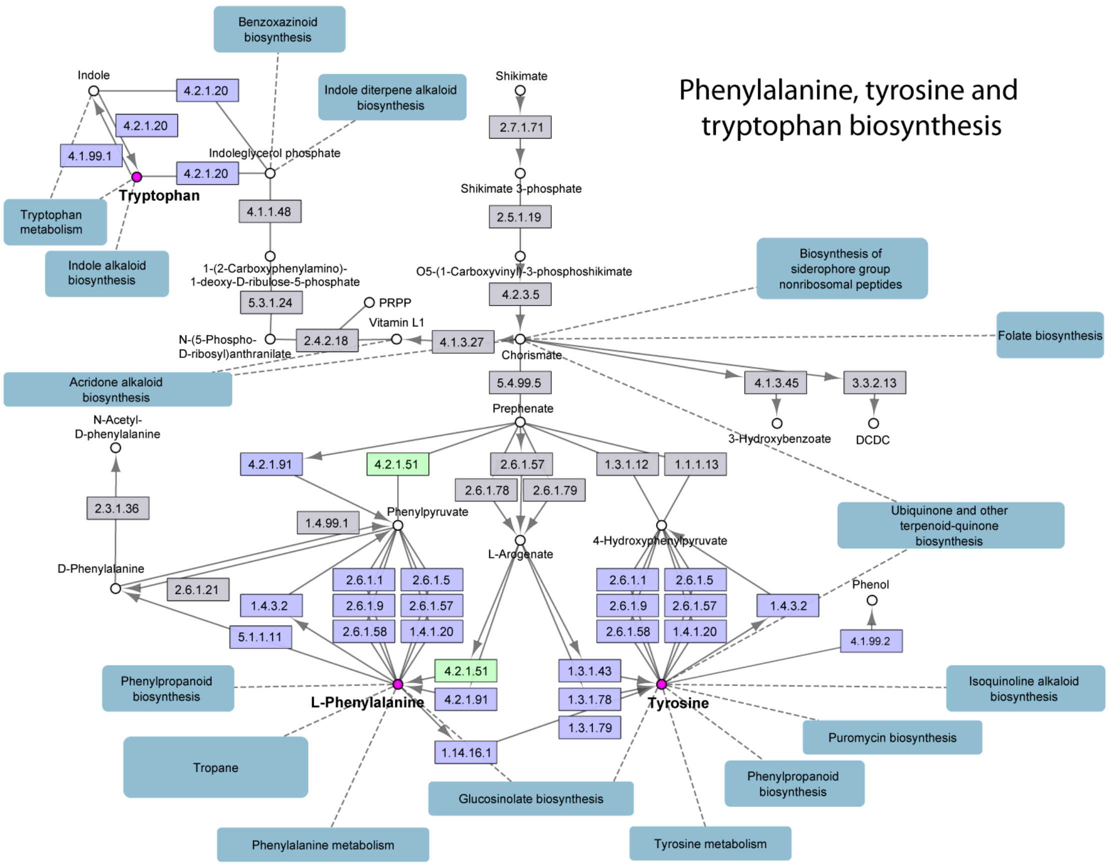

Figure B. Monoamine precursor biosynthesis pathways.

Visualization of a selection of the pathway maps of 'Phenylalanine, tyrosine and tryptophan biosynthesis' according to KEGG [2], created in Cytoscape [3] with the KEGGscape app [4] by combining various maps (ko00400, ko00360, ko00350 and ko00380) into one representation. Nodes represent compounds, pink nodes represent one of our compounds of interest: phenylalanine, tyrosine or tryptophan. Boxes represent enzyme functions/reactions, numbers shown inside are enzyme (EC) numbers. Boxes in blue correspond to candidates that were a priory selected for our function analysis by PICRUSt, gray boxes were excluded from analysis. Arrows link the conversion of one compound to another, facilitated by an enzyme function/reaction. Dashed lines link compounds to other KEGG pathways.

The EC number with the green box (EC:4.2.1.51; K01713) represents the enzyme cyclohexadienyl dehydratase and is the one candidate reaction showing a diagnosis effect (see main **Figure 4**). Cyclohexadienyl dehydratase (also known as arogenate dehydratase) is involved in the synthesis of phenylalanine by two different routes: firstly, by directly converting arogenate to phenylalanine [5], secondly, by catalyzing the reaction of prephenate to phenylpyruvate [6]. Phenylpyruvate is then transaminated to phenylalanine by phenylpyruvate aminotransferase [7, 8].

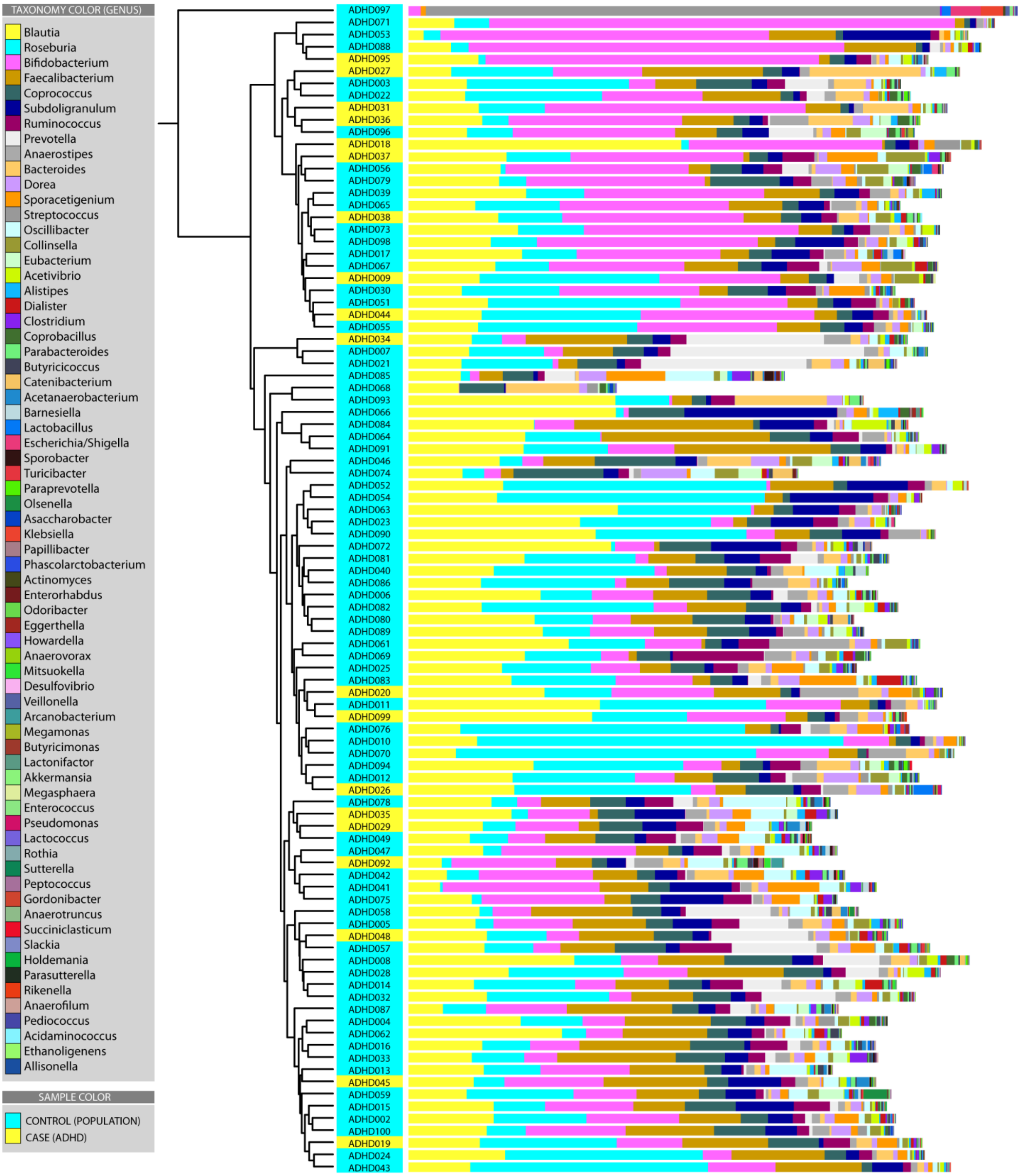

Figure C. Intestinal microbiome composition.

Clustering of 96 samples based on intestinal microbial composition on the genus level: 77 controls (population; in blue) and 19 cases (ADHD; in yellow). The composition is displayed as absolute abundance, which is the number of reads assigned to a genus. Color bars represent absolute abundance of these bacterial genera as determined by 454 pyrosequencing. Hierarchical UPGMA clustering (Unweighted Pair Group Method with Arithmetic Mean) was performed with weighed UniFrac as distance measure, as implemented in QIIME 1.2. The figure was generated with the interactive tree of life (iTOL) program [9].

Text A: Cohorts

1. NeuroIMAGE II cohort

NeuroIMAGE II is a follow-up of NeuroIMAGE [10], which is the Dutch follow-up of the IMAGE study on ADHD performed between 2003-2006 (as described previously in [11, 12]). Families with at least one child with combined subtype ADHD and at least one biological sibling (regardless of ADHD diagnosis) were recruited, in addition to control families with at least one child, with no formal or suspected ADHD diagnosis in any of the first-degree family members.

Inclusion criteria for participants were: between 8-30 years, of European Caucasian descent, an IQ ≥ 70, and no diagnosis of autism (as determined in the preceding IMAGE project [13] using criteria of the DSM-IV), epilepsy, general learning difficulties, brain disorders and known genetic disorders (such as Fragile X syndrome or Down syndrome), and no contraindication to MRI scanning (e.g. implanted metal or medical devices, or possible pregnancy). Participants were asked to withhold use of psychoactive drugs for 48 hours before measurement.

The NeuroIMAGE follow-up study had a comprehensive assessment protocol encompassing questionnaires, a diagnostic interview and several neurocognitive measures from all family members, as well as an extensive MRI scanning protocol in participants. During the testing day, participants were motivated with short breaks, and at the end of the day, they received a reward of €50,-. Participants gave written informed consent (and their parents when <18 years old) and the study was approved by the regional medical ethics committee.

To determine ADHD diagnoses at the follow-up measurement in the NeuroIMAGE study, all participants in the study were similarly assessed using a semi-structured diagnostic interview. For participants using medication, ratings were done of children's functioning off medication. All participants were administered the Dutch translation of the Kiddie Schedule for Affective Disorders and Schizophrenia (K-SADS) Present and Lifetime Version [14], carried out by trained professionals. Both the parents and the child, if ≥ 12 years old, were interviewed separately and were initially only administered the ADHD screening interview. Participants with elevated scores on any of the screen items were administered the full ADHD section. Participants with a symptom count of ≥ 6 symptoms of either hyperactive/impulsive behaviour or inattentive behaviour were diagnosed with ADHD, provided they: a) met the DSM-IV criteria (American Psychiatric Association, 2000) for pervasiveness and impact of the disorder (measures derived from the K-SADS), b) showed an age of onset before 12 (following the proposed changes for the DSM-V; see [15]), derived from the K-SADS. Criteria were slightly adapted for young adults (≥ 18 years), such that a symptom count of 5 symptoms on either hyperactive/impulsive or inattentive scale was sufficient for a diagnosis [16]. Young adults were considered unaffected

when they received ≤ 2 symptoms on the combined symptom counts. Inconsistent cases were evaluated by a team of trained experts (consisting psychiatrist JB and 8 psychologists), in order to derive a consensus diagnosis.

2. BIG cohort

The remaining microbiome participants (n=39) were selected from the Brain Imaging Genetics (BIG) study. The study sample consisted of healthy adult volunteers taking part in the diverse studies conducted at the Donders Institute for Brain, Cognition and Behaviour in Nijmegen, The Netherlands [17]. Participants of the BIG cohort were of Caucasian origin, between 18 and 36 years of age and right-handed. No diagnostic assessment was performed, but participants had no self-reported neurological or psychiatric history. Participants gave written informed consent and the study was approved by the regional medical ethics committee.

3. Inclusion criteria microbiome part

Participants of the BIG cohort (control subjects) could take part in the microbiome study if they were older than 18 years, not pregnant (for women), did not have chronic or acute diseases at the time of assessment, did not use chronic or acute medication (including antibiotics) during the last month before the study, and were of Western-European descent. Retrospectively, we discovered that one participant used medication and one participant was not from Western-European descent but from northern-African descent. Our analysis of differences in gut microbiome metabolic potential (with PICRUSt) did not change qualitatively when excluding these two controls; i.e. the cyclohexadienyl dehydratase (CDT) difference between ADHD and controls (see main **Results**) remained significant (p=0.044). None of the fMRI analyses included BIG controls.

Text B: fMRI parameters and analyses

1. fMRI data acquisition

Whole-brain imaging was performed on a 1.5T MR scanner (Magnetom Avanto, Siemens Medical Systems, Erlangen, Germany). BOLD sensitive functional images were acquired using a T2*-weighted multi-echo EPI sequence (TR: 2.67 s; TEs for 5 echoes: 7.7 ms, 17.3 ms, 27.0 ms, 36.6 ms, and 46.3 ms). We used a multi-echo EPI sequence to reduce image distortion and increase BOLD sensitivity in our regions of interest which are typically affected by strong susceptibility artifacts, such as the ventral striatum [18]. One volume consisted of 37 axial slices (voxel size: 3.5 x 3.5 x 3.0 mm3; interslice gap: 0.5 mm; field of view: 224 mm; flip angle: 90 degrees). All images were acquired in a single run comprising 20 min. Visual stimuli were projected on a screen and were viewed through a mirror attached to the head coil. In addition to the acquisition of functional images, a high-resolution T1-weighted MP- RAGE anatomical scan was obtained (176 sagittal slices, TR: 2.73 s, TE: 2.95 ms, voxel size: 1.0 x 1.0 x 1.0 mm3, field of view: 256 mm).

2. fMRI preprocessing

Echo-time (TE) weighted summation was then used to combine all five echoes into a single data set. Realignment parameters were estimated from the combined TE-images using a least squares approach and a 6 parameter (rigid body) spatial transformation (Friston *et al.*, 1995). During subsequent slice timing correction, the time-series for each voxel were realigned temporally to acquisition of the middle slice. Anatomical images were spatially co-registered to the mean of the functional images and segmented using a unified segmentation approach as implemented in the VBM8 toolbox in SPM (http://www.neuro.uni-jena.de/vbm/). The resulting transformation matrix and warp field were then used to normalize the anatomical and functional images into the common MNI152 reference space. Normalized images were spatially smoothed with an isotropic 6 mm full-width-half-maximum (FWHM) Gaussian kernel.

3. fMRI statistical analysis

The first level model included 2 regressors for reward cues (high, low) and 4 regressors for the targets (high_congruent, high_incongruent, low_congruent, low_incongruent). All regressors of interest were modeled as an impulse response function (duration = 0) convolved with a canonical haemodynamic response function. Regressors of non-interest included: the 15-second breaks, missed targets (no button response), and 24 motion parameters to optimally control for motion effects (i.e. the linear and quadratic effects of x, y, z, pitch, roll, and yaw movement). Functional scans were high-pass filtered (128 seconds) to remove low-frequency confounds such as scanner drifts. Parameter estimates for all regressors were

obtained by maximum-likelihood estimation, modeling temporal autocorrelation as an AR(1) process. Contrast images from the first level were entered into second level random effects analyses. To further account for motion, we calculated a summary motion score for every subject, as the sum of the root-mean-square value of subjects' framewise-displacement parameters (x, y, z in mm & pitch, roll, and yaw in degrees) [19]. This score was greater for ADHD cases than controls ($t(85)=-2.66$, $p=.009$), which is why we added this individual summary motion score in all second level analyses as covariate of non-interest.

4. Region of Interest (ROI)

As our ventral striatal ROI, we used the anatomically-defined bilateral nucleus accumbens region from the Hammersmith atlas (www.brain-development.org [20]). We extracted the mean beta-weights for every participant with MarsBar [21]. The regionally averaged betas were used to assess the effects of ADHD diagnosis on reward anticipation responses using an independent-sample t-test in SPSS version 22 (IBM Corp. IBM SPSS Statistics, Armonk, New York, USA).

5. Psychostimulant predictor in multiple regression

We ran a multiple regression analysis with reward anticipation responses in the ventral striatum as dependent. In addition to the functional microbiome measure, one of the other predictors was long-term medication use. For this predictor, we calculated the duration of psychostimulant use minus the period not using psychostimulants anymore (if any), and multiplied this with the instant dose. For the sustained-release Concerta, we calculated the instant dose by multiplying the daily dose with 0.278, and added this to the immediate-release Ritalin dose (if any). This duration x dose amount was used as the medication predictor in the multiple regression analysis. Out of the 28 subjects in this multiple regression analysis, six were (n=5) or had been (n=1) using medication for ADHD; all six were using Ritalin and/or Concerta.

Text C: Microbiome sequencing and analyses

1. 16S marker gene amplification, sequencing and data acquisition

According to Jaeggi and colleagues [22], the V3-V6 region of the 16S rRNA gene was amplified by PCR using the following universal primers: (i) forward primer, 5'-*CCATCTCATCCCTGCGTGTCTCCGACTAG*NNNNNN**ACTCCTACGGGAGGCAGCAG**-3' (the italicised sequence is the 454 Life Sciences primer A, and the bold sequence is the broadly conserved bacterial primer 338F; NNNNNN designates the sample-specific six-base barcode used to tag each PCR product, see **S1 Table**); (ii) reverse primer 5'-*CCTATCCCCTGTGTGCCTTGGCAGTCTCAG***CRRCACGAGCTGACGAC**-3' (the italicised sequence is the 454 Life Sciences primer B, and the bold sequence is the broadly conserved bacterial primer 1061R).

The PCR amplification mixture contained: 1 µL faecal DNA, 1 µL bar-coded forward primer, 15 µL master mix (1 µL KOD Hot Start DNA Polymerase (1 U/µL; Novagen, Madison, WI, USA), 5 µL KOD-buffer (10×), 3 µL MgSO4 (25 mM), 5 µL dNTP mix (2 mM each), 1 µL (10 µM) of reverse primer) and 33 µL sterile water (total volume 50 µL). PCR conditions were: 95°C for 2 min. followed by 35 cycles of 95°C for 20 sec., 55°C for 10 sec., and 70°C for 15 sec. The approximately 750 bp PCR amplicon was subsequently purified using the MSB Spin PCRapace kit (Invitek, Hayward, CA) and the concentration was checked with a Nanodrop 1000 spectrophotometer (Thermo Scientific, Thermo Fisher Scientific, Waltham, MA). A composite sample for pyrosequencing was prepared by pooling 200 ng of these purified PCR products of each sample. The pooled sample was purified using the Purelink PCR Purification kit (Invitrogen, Thermo Fisher Scientific, Waltham, MA), with high-cut-off binding buffer B3, and submitted for pyrosequencing of the V3-V4 region of the 16S rRNA gene on the 454 Life Sciences GS-FLX platform using Titanium sequencing chemistry (GATC-Biotech, Germany).

2. Microbiome sequencing data analysis and bioinformatics workflow

Reads were filtered for chimeric sequences using the UCHIME algorithm version 4 [23]. Hierarchical clustering of samples was performed using UPGMA with weighted UniFrac as a distance measure as implemented in QIIME 1.2. Figures resulting from these clustering analyses were generated using the interactive tree of life (iTOL) tool [9]. The Ribosomal Database Project classifier version 2.3 was performed for taxonomic classification of the sequence reads [24]. Alpha diversity metrics (PD whole tree, Chao1, Observed Species and Shannon) were calculated by bootstrapping 1126 reads per sample, and taking the average over four trials. For visualization of the differential microbiome, Cytoscape software version 3.1 [3] was used together with in-house developed Python scripts for generating the appropriate input data deriving from the QIIME analysis.

Note that due to technical limitations in the resolution of 16S marker gene sequencing, OTU (Operational Taxonomic Unit) calling on the level of species should be interpreted with caution (**S2 Table**, **Figure 3**).

3. Microbiome-derived function prediction

We predicted the presence of Kyoto Encyclopedia of Genes and Genomes (KEGG) Orthologs (K numbers; note that K numbers can represent orthologs with multiple enzymatic functions and vice versa) [25] and subsequent functional and metabolic pathways using PICRUSt (Phylogenetic Investigation of Communities by Reconstruction of Unobserved States; version 1.0.0) [26]. PICRUSt requires closed-reference OTU picking in QIIME, for which the Greengenes reference collection version 13.5 (May 2013) was used [27]. For additional calculation of relative abundances of pathways and KEGG Orthologs (i.e. the candidates), and for downstream statistics, in-house Python scripts were used.

Text D: Additional information microbiome

1. Gut microbiome sample characteristics

The 16S marker gene 454 pyrosequencing run was performed in a total of 96 samples and yielded a total of 363,461 high quality reads with good sequencing depth; roughly 80% of the reads could be traced back to a specific bacterial genus. No apparent differences in either alpha or beta diversity between the microbiome of ADHD cases and controls could be observed (see main **Table 1** for Shannon and Chao index; beta diversity not reported). For further details, see **S1 Table**.

2. Age-matched sub-sample of ADHD cases and controls

Table. Age-matched sub-sample

Pair *	Sample ID	Age (years)	Gender	Diagnosis
Pair 1	ADHD014	15	female	CONTROL
	ADHD026	15	female	CASE
Pair 2	ADHD041	16	male	CONTROL
	ADHD020	16	female	CASE
Pair 3	ADHD008	17	female	CONTROL
	ADHD037	17	female	CASE
Pair 4	ADHD002	18	female	CONTROL
	ADHD092	18	female	CASE
Pair 5	ADHD096	19	male	CONTROL
	ADHD018	19	female	CASE
Pair 6	ADHD003	20	male	CONTROL
	ADHD029	20	male	CASE
Pair 7	ADHD090	20	male	CONTROL
	ADHD099	20	male	CASE
Pair 8	ADHD093	20	female	CONTROL
	ADHD048	20	male	CASE
Pair 9	ADHD052	20	female	CONTROL
	ADHD044	20	female	CASE
Pair 10	ADHD024	21	male	CONTROL
	ADHD038	21	male	CASE
Pair 11	ADHD021	21	male	CONTROL
	ADHD035	21	male	CASE
Pair 12	ADHD040	21	female	CONTROL
	ADHD034	21	male	CASE
Pair 13	ADHD071	22	male	CONTROL
	ADHD045	22	male	CASE
Pair 14	ADHD028	23	male	CONTROL
	ADHD019	23	male	CASE
Pair 15	ADHD047	25	female	CONTROL
	ADHD095	25	male	CASE

* age-matched pairs; four ADHD subjects did not have a matching control subject

3. Gut microbiome metabolic potential: logistic regression including age

A logistic regression analysis on all 15 present candidate reactions was performed to assess the effect of age in our cohort. Age was found to have a significant effect in all candidate reactions when including all controls; p-values < 0.05). The logistic regression model used case control status as dependent variable and age, age^2, gender and the relative abundance of the candidate reactions as covariates (age^2 was added in order to account better for the age effect). For the analysis with the complete sample, reaction K01713 showed suggestive evidence for association (p-value=0.070, OR=1.921, 95% confidence interval (CI) (0.948-3.895); n=96). After removing the BIG controls the association between CDT (K01713) and ADHD risk was significant (p-value=0.024, OR=2.786, 95% CI (1.144-6.785); n=57).

4. Relation between taxa and reactions

To investigate which taxa from the above analysis contributed the most to the phenylalanine biosynthesis reaction that differed significantly between ADHD cases and controls, we repeated the functional (PICRUSt) analysis only on the five candidate taxa which differed the most between ADHD and controls: Clostridiales (order), Rikenellaceae (family), Porphyromonadaceae (family), *Bifidobacterium* (genus) and *Eggerthella* (genus) (main **Figure 3**). For this selection of taxa, we again observed a similar increase in ADHD of cyclohexadienyl dehydratase (K01713) as compared to the total microbiome (on average, 150% of control relative abundance; p = 0.038, BF corrected). Strikingly, we found that the cyclohexadienyl dehydratase ortholog (K01713) was solely present in Actinobacteria and its descending genus *Bifidobacterium*, not in any of the four candidate taxa. Specifically, in an additional PICRUSt functional analysis selecting only the genus *Bifidobacterium*, we observed that the total number of absolute counts for K01713 for the control and ADHD group combined was the same number when examining the analysis for the above described combined selection of five candidate taxa (16,329; representing an average relative abundance of 0.0044 %); and more strikingly, accounting for 99.9% of the PICRUSt predicted CDT counts in the entire microbiome (**S2 Table**).

Consequently, in pursue of the question which taxon or taxa were contributing the most to the presence of cyclohexadienyl dehydratase in the gut microbiomes, we did a Spearman correlation analysis on K01713 with all separate taxa on all hierarchical levels. Interestingly, a very strong positive correlation was observed for Actinobacteria (phylum/class; rho = 0.97) and *Bifidobacterium* (see **figure below**) (genus, but also for its family and order; rho = 0.98); all surviving multiple testing correction. Furthermore, a strong negative correlation was observed for Firmicutes (phylum; rho = -0.80) and Clostridiales (order; rho = -0.75); also surviving multiple testing correction (data not shown). However, these latter effects can probably best be explained by the fact that a large increase in relative

abundance for Actinobacteria and its descending taxonomies consequently lowers all other phyla in relative abundance, which reasonably affects Firmicutes and its descending taxonomies the most, as Firmicutes are by far the most represented bacteria in the gut microbiomes of our subjects (on average, 79.80% in the control group; main **Figure 3; S2 Table**).

The idea that the negative correlations between Firmicutes/Clostridiales and CDT (K01713) were not independent from the positive correlation between CDT and *Bifidobacterium* was confirmed by a multiple regression analysis. We took relative abundance of the cyclohexadienyl dehydratase reaction (K01713) as dependent and used the relative abundances of the same five candidate taxa as above (Clostridiales (order), Rikenellaceae (family), Porphyromonadaceae (family), *Bifidobacterium* (genus) and *Eggerthella* (genus)) as predictors. The overall model was significant ($F(4,91)=298.7$, $p < 0.001$, adjusted R square = 0.943), which is in line with the fact that the differences in reactions were determined based on the differences in taxa. However, only the genus *Bifidobacterium* showed a strong positive association with the phenylalanine biosynthesis reaction (standardized beta = 0.94, $t=25.3$, $p < 0.001$). The other candidate taxa, including Clostridiales (phylum Firmicutes), did not show significant associations with the cyclohexadienyl dehydratase reaction (all standardized betas < 0.05 and > -0.05 and $p > 0.1$) in the multiple regression model. Therefore, differences in relative abundance of genus *Bifidobacterium* exclusively contributed to the observed differences in the phenylalanine biosynthesis pathway.

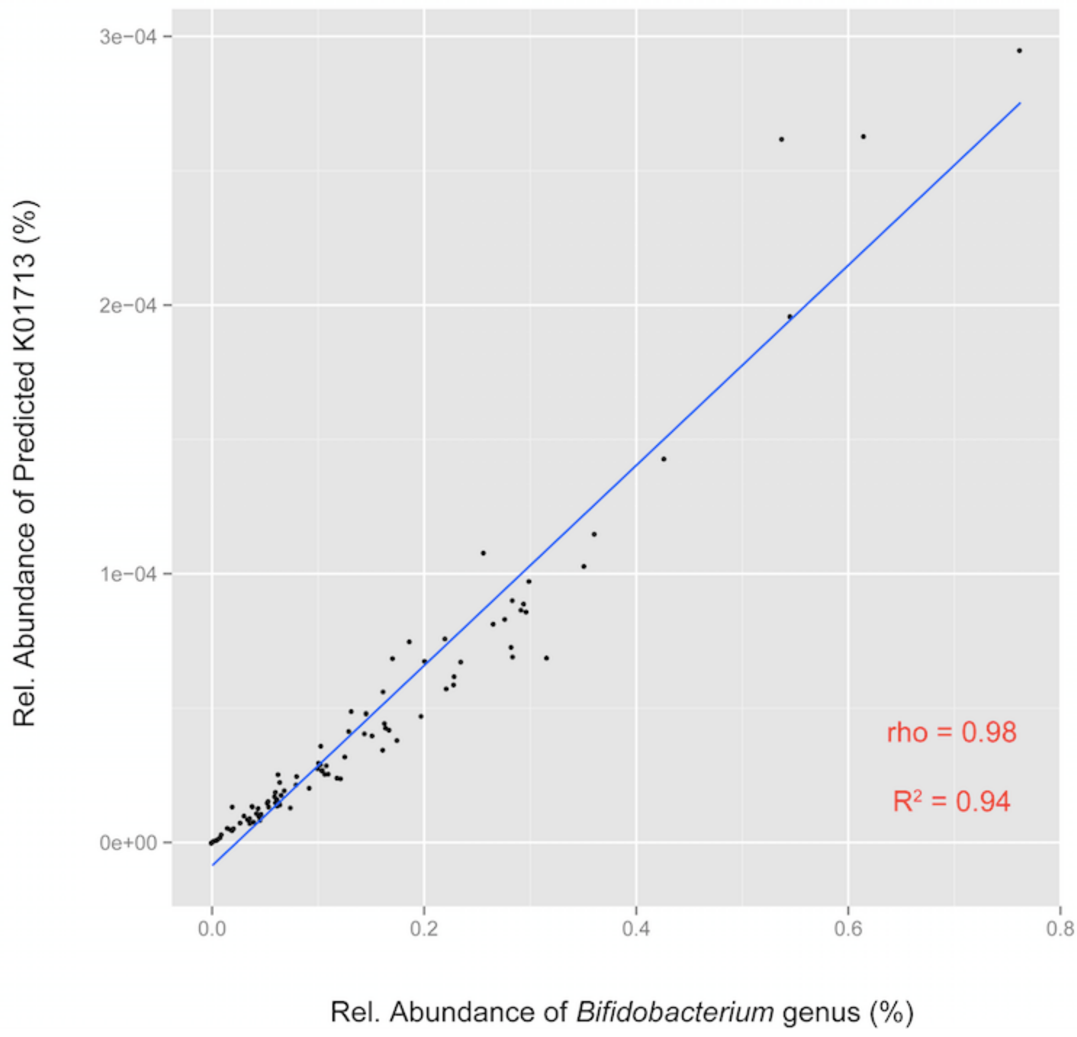

Figure. Spearman correlation of the relative abundance levels of predicted cyclohexadienyl dehydratase (CDT; KEGG Ortholog K01713; EC:4.2.1.9151) to the relative levels of *Bifidobacterium* genus according to 454 pyrosequencing, calculated over all 96 ADHD and control samples in the cohort ($p < 0.001$, Bonferroni corrected).

Text E: fMRI main effects of reward anticipation

Table. MNI stereotactic coordinates of local BOLD maxima (max. 3) in clusters significant for the main effect of reward anticipation (15 > 1 cent cues) at pFWE<0.05 (cluster level).

		cluster statistics		local maxima	
region	side	p(FWE)	size	T-value	x,y,z
**n=87**					
superior occipital gyrus	R	<.001	39442	10.72	15 -91 3
calcarine sulcus	L			10.27	-12 -93 -0
precentral gyrus	L	<.001	30729	7.83	-26 -9 46
supplementary motor area	L			7.58	-6 -1 49
supplementary motor area	R			7.19	9 6 46
putamen	R	<.001	8824	7.34	18 12 -0
caudate	L			6.88	-9 11 -3
middle frontal gyrus	L	<.001	1802	6.25	-38 44 19
middle frontal gyrus	R	<.001	735	5.28	36 44 25
superior temporal gyrus	R	0.016	320	4.18	57 8 -3
**n=28**					
superior occipital gyrus	R	<.001	3658	7.53	20 -93 7
inferior occipital gyrus	R			6.66	33 -81 -6
lingual gyrus	R			6.37	20 -94 -6
calcarine sulcus	L	<.001	4474	6.84	-12 -88 -0
middle occipital gyrus	L			6.71	-27 -93 3
inferior occipital gyrus	L			6.06	-39 -85 -8
precentral gyrus	L	<.001	9571	6.71	-26 -7 48
inferior parietal lobe	L			6.16	-45 -31 36
putamen	L	<.001	1027	6.65	-12 9 -9
caudate	L			5.46	-8 0 -2
precentral gyrus	R	<.001	2063	6.02	35 -16 69
pallidum	R	<.001	1013	5.68	18 15 1
putamen	R			4.73	20 20 -6
cerebellum	R	0.004	385	5.65	14 -66 -21
superior occipital gyrus	L	<.001	1166	5.62	-24 -70 30
precuneus	L			4.74	-11 -72 48
superior parietal lobe	L			4.57	-29 -64 49
cerebellum	L	0.007	352	5.38	-27 -51 -29
fusiform gyrus	L			4.25	-35 -52 -20
cerebellum	R	<.001	941	5.31	36 -48 -30
middle cingulate gyrus	L	0.002	443	5.01	-3 -27 30
middle cingulate gyrus	R			4.53	5 -24 34
posterior cingulate gyrus	L			4.36	-3 -39 25
cerebellum	R	<.001	671	4.96	18 -68 -50
superior parietal lobe	R	0.001	465	4.92	23 -49 52
middle frontal gyrus	L	0.004	388	4.89	-32 35 36
middle frontal gyrus	R	0.049	231	4.61	33 54 4
superior orbitofrontal gyrus	R			4.47	24 54 -2

Text F: References in S1 Appendix

1. Aarts E, Wallace DL, Dang LC, Jagust WJ, Cools R, D'Esposito M. Dopamine and the cognitive downside of a promised bonus. Psychological science. 2014;25(4):1003-9. Epub 2014/02/15. doi: 10.1177/0956797613517240
0956797613517240 [pii]. PubMed PMID: 24525265; PubMed Central PMCID: PMC4163051.

2. Kanehisa M, Goto S. KEGG: kyoto encyclopedia of genes and genomes. Nucleic acids research. 2000;28(1):27-30. PubMed PMID: 10592173; PubMed Central PMCID: PMC102409.

3. Shannon P, Markiel A, Ozier O, Baliga NS, Wang JT, Ramage D, et al. Cytoscape: a software environment for integrated models of biomolecular interaction networks. Genome research. 2003;13(11):2498-504. doi: 10.1101/gr.1239303. PubMed PMID: 14597658; PubMed Central PMCID: PMC403769.

4. Nishida K, Ono K, Kanaya S, Takahashi K. KEGGscape: a Cytoscape app for pathway data integration. F1000Research. 2014;3:144. doi: 10.12688/f1000research.4524.1. PubMed PMID: 25177485; PubMed Central PMCID: PMC4141640.

5. Fischer R, Jensen R. Arogenate dehydratase. Methods in enzymology. 1987;142:495-502. PubMed PMID: 3600377.

6. Cotton RG, Gibson F. The Biosynthesis of Phenylalanine and Tyrosine; Enzymes Converting Chorismic Acid into Prephenic Acid and Their Relationships to Prephenate Dehydratase and Prephenate Dehydrogenase. Biochimica et biophysica acta. 1965;100:76-88. PubMed PMID: 14323651.

7. Obmolova G, Teplyakov A, Xia T, Jensen R. Preliminary crystallographic study of cyclohexadienyl dehydratase from Pseudomonas aeruginosa. Journal of molecular biology. 1993;232(3):992-4. doi: 10.1006/jmbi.1993.1446. PubMed PMID: 8355283.

8. Yoo H, Widhalm JR, Qian Y, Maeda H, Cooper BR, Jannasch AS, et al. An alternative pathway contributes to phenylalanine biosynthesis in plants via a cytosolic tyrosine:phenylpyruvate aminotransferase. Nature communications. 2013;4:2833. doi: 10.1038/ncomms3833. PubMed PMID: 24270997.

9. Letunic I, Bork P. Interactive Tree Of Life (iTOL): an online tool for phylogenetic tree display and annotation. Bioinformatics. 2007;23(1):127-8. doi: 10.1093/bioinformatics/btl529. PubMed PMID: 17050570.

10. von Rhein D, Mennes M, van Ewijk H, Groenman AP, Zwiers MP, Oosterlaan J, et al. The NeuroIMAGE study: a prospective phenotypic, cognitive, genetic and MRI study in children with attention-deficit/hyperactivity disorder. Design and descriptives. European child & adolescent psychiatry. 2015;24(3):265-81. doi: 10.1007/s00787-014-0573-4. PubMed PMID: 25012461.

11. Rommelse NN, Arias-Vasquez A, Altink ME, Buschgens CJ, Fliers E, Asherson P, et al. Neuropsychological endophenotype approach to genome-wide linkage analysis identifies susceptibility loci for ADHD on 2q21.1 and 13q12.11. American journal of human genetics. 2008;83(1):99-105. doi: 10.1016/j.ajhg.2008.06.006. PubMed PMID: 18599010; PubMed Central PMCID: PMC2443830.

12. Kuntsi J, Neale BM, Chen W, Faraone SV, Asherson P. The IMAGE project: methodological issues for the molecular genetic analysis of ADHD. Behavioral and brain functions : BBF. 2006;2:27. doi: 10.1186/1744-9081-2-27. PubMed PMID: 16887023; PubMed Central PMCID: PMC1559631.

13. Brookes K, Xu X, Chen W, Zhou K, Neale B, Lowe N, et al. The analysis of 51 genes in DSM-IV combined type attention deficit hyperactivity disorder: association signals in DRD4, DAT1 and 16 other genes. Molecular psychiatry. 2006;11(10):934-53. PubMed PMID: 16894395.

14. Kaufman J, Birmaher B, Brent D, Rao U, Flynn C, Moreci P, et al. Schedule for Affective Disorders and Schizophrenia for School-Age Children-Present and Lifetime Version (K-SADS-PL): initial reliability and validity data. Journal of the American Academy of Child and Adolescent Psychiatry. 1997;36(7):980-8. doi: 10.1097/00004583-199707000-00021. PubMed PMID: 9204677.

15. Polanczyk G, Caspi A, Houts R, Kollins SH, Rohde LA, Moffitt TE. Implications of extending the ADHD age-of-onset criterion to age 12: results from a prospectively studied birth cohort. Journal of the American Academy of Child and Adolescent Psychiatry. 2010;49(3):210-6. PubMed PMID: 20410710.

16. Kooij JJ, Buitelaar JK, van den Oord EJ, Furer JW, Rijnders CA, Hodiamont PP. Internal and external validity of attention-deficit hyperactivity disorder in a population-based sample of adults. Psychological medicine. 2005;35(6):817-27. PubMed PMID: 15997602.

17. Franke B, Vasquez AA, Veltman JA, Brunner HG, Rijpkema M, Fernandez G. Genetic variation in CACNA1C, a gene associated with bipolar disorder, influences brainstem rather than gray matter volume in healthy individuals. Biological psychiatry. 2010;68(6):586-8. doi: 10.1016/j.biopsych.2010.05.037. PubMed PMID: 20638048.

18. Poser BA, Versluis MJ, Hoogduin JM, Norris DG. BOLD contrast sensitivity enhancement and artifact reduction with multiecho EPI: parallel-acquired inhomogeneity-desensitized fMRI. Magn Reson Med. 2006;55(6):1227-35. Epub 2006/05/09. doi: 10.1002/mrm.20900. PubMed PMID: 16680688.

19. Jenkinson M, Bannister P, Brady M, Smith S. Improved optimization for the robust and accurate linear registration and motion correction of brain images. NeuroImage. 2002;17(2):825-41. PubMed PMID: 12377157.

20. Hammers A, Allom R, Koepp MJ, Free SL, Myers R, Lemieux L, et al. Three-dimensional maximum probability atlas of the human brain, with particular reference to the temporal lobe. Human brain mapping. 2003;19(4):224-47. doi: 10.1002/hbm.10123. PubMed PMID: 12874777.

21. Brett M, Anton JL, Valabregue R, Poline JB, editors. Region of interest analysis using an SPM toolbox. 8th International Conference on Functional Mapping of the Human Brain; 2002; Sendai, Japan: NeuroImage.

22. Jaeggi T, Kortman GA, Moretti D, Chassard C, Holding P, Dostal A, et al. Iron fortification adversely affects the gut microbiome, increases pathogen abundance and induces intestinal inflammation in Kenyan infants. Gut. 2015;64(5):731-42. doi: 10.1136/gutjnl-2014-307720. PubMed PMID: 25143342.

23. Edgar RC, Haas BJ, Clemente JC, Quince C, Knight R. UCHIME improves sensitivity and speed of chimera detection. Bioinformatics. 2011;27(16):2194-200. doi: 10.1093/bioinformatics/btr381. PubMed PMID: 21700674; PubMed Central PMCID: PMC3150044.

24. Cole JR, Wang Q, Cardenas E, Fish J, Chai B, Farris RJ, et al. The Ribosomal Database Project: improved alignments and new tools for rRNA analysis. Nucleic acids research. 2009;37(Database issue):D141-5. doi: 10.1093/nar/gkn879. PubMed PMID: 19004872; PubMed Central PMCID: PMC2686447.

25. Kanehisa M, Goto S, Sato Y, Kawashima M, Furumichi M, Tanabe M. Data, information, knowledge and principle: back to metabolism in KEGG. Nucleic acids research. 2014;42(Database issue):D199-205. doi: 10.1093/nar/gkt1076. PubMed PMID: 24214961; PubMed Central PMCID: PMC3965122.

26. Langille MG, Zaneveld J, Caporaso JG, McDonald D, Knights D, Reyes JA, et al. Predictive functional profiling of microbial communities using 16S rRNA marker gene sequences. Nature biotechnology. 2013;31(9):814-21. doi: 10.1038/nbt.2676. PubMed PMID: 23975157; PubMed Central PMCID: PMC3819121.

27. DeSantis TZ, Hugenholtz P, Larsen N, Rojas M, Brodie EL, Keller K, et al. Greengenes, a chimera-checked 16S rRNA gene database and workbench compatible with ARB. Applied and environmental microbiology. 2006;72(7):5069-72. doi: 10.1128/AEM.03006-05. PubMed PMID: 16820507; PubMed Central PMCID: PMC1489311.
